# Impacts of extreme heat on emergency medical service calls in King County, Washington, 2007–2012: relative risk and time series analyses of basic and advanced life support

**DOI:** 10.1186/s12940-016-0109-0

**Published:** 2016-01-28

**Authors:** Miriam M. Calkins, Tania Busch Isaksen, Benjamin A. Stubbs, Michael G. Yost, Richard A. Fenske

**Affiliations:** Department of Environmental and Occupational Health Sciences, School of Public Health, University of Washington, 1959 NE Pacific St., P.O. Box 237234, Seattle, WA 98195 USA; Emergency Medical Services Division, Seattle and King County Department of Public Health, 401 5th Ave, Suite 1200, Seattle, WA 98104 USA

**Keywords:** Extreme heat, Climate change, Emergency medical services, Ambulance, Relative risk, Time series

## Abstract

**Background:**

Exposure to excessive heat kills more people than any other weather-related phenomenon, aggravates chronic diseases, and causes direct heat illness. Strong associations between extreme heat and health have been identified through increased mortality and hospitalizations and there is growing evidence demonstrating increased emergency department visits and demand for emergency medical services (EMS). The purpose of this study is to build on an existing regional assessment of mortality and hospitalizations by analyzing EMS demand associated with extreme heat, using calls as a health metric, in King County, Washington (WA), for a 6-year period.

**Methods:**

Relative-risk and time series analyses were used to characterize the association between heat and EMS calls for May 1 through September 30 of each year for 2007–2012. Two EMS categories, basic life support (BLS) and advanced life support (ALS), were analyzed for the effects of heat on health outcomes and transportation volume, stratified by age. Extreme heat was model-derived as the 95th (29.7 **°**C) and 99th (36.7 °C) percentile of average county-wide maximum daily humidex for BLS and ALS calls respectively.

**Results:**

Relative-risk analyses revealed an 8 % (95 % CI: 6–9 %) increase in BLS calls, and a 14 % (95 % CI: 9–20 %) increase in ALS calls, on a heat day (29.7 and 36.7 °C humidex, respectively) versus a non-heat day for all ages, all causes. Time series analyses found a 6.6 % increase in BLS calls, and a 3.8 % increase in ALS calls, per unit-humidex increase above the optimum threshold, 40.7 and 39.7 °C humidex respectively. Increases in “no” and “any” transportation were found in both relative risk and time series analyses. Analysis by age category identified significant results for all age groups, with the 15–44 and 45–64 year old age groups showing some of the highest and most frequent increases across health conditions. Multiple specific health conditions were associated with increased risk of an EMS call including abdominal/genito-urinary, alcohol/drug, anaphylaxis/allergy, cardiovascular, metabolic/endocrine, diabetes, neurological, heat illness and dehydration, and psychological conditions.

**Conclusions:**

Extreme heat increases the risk of EMS calls in King County, WA, with effects demonstrated in relatively younger populations and more health conditions than those identified in previous analyses.

## Background

According to the Centers for Disease Control and Prevention (CDC), exposure to excessive heat kills more people than any other weather-related phenomenon, aggravates chronic diseases, and causes direct heat illness [1]. Strong associations between extreme heat and health have been identified in the literature for increased mortality [2–10] as well as hospitalizations [7, 10–13] and there is growing evidence demonstrating increased emergency department visits [12, 14, 15] and demand for emergency medical services (EMS) [7–9, 16–24]. With high confidence in projected increases in the frequency and duration of extreme heat attributable to climate change [25, 26], a thorough understanding of the impacts of extreme heat on human health and the public health system is vital to efficient prevention, management, and mitigation of long-term consequences.

The existing literature describing the relationship of extreme heat and EMS calls consistently reports significant increases in risk, despite inconsistent definitions and measures of heat. Most of these studies have occurred outside the United States, including Australia [7, 8, 16], Canada [17, 18], Italy [19], Japan [20], and Switzerland [9]. Three studies have focused on U.S. cities—Boston [21], Chicago [22] and Phoenix [23]; a fourth study compared Chicago with Phoenix [24]. Many studies examined EMS dispatches for a single health condition, including heat-related dispatch or illness [4, 18, 22, 24], pre-hospital electrocardiograms (ECG) [19], and heatstroke [20], while others only reported total call volume. Other related investigations include transportation volume [21] and identification of additional extreme heat-related factors driving EMS needs, such as hazards resulting from multi-system failures (e.g., power-outages) [27]. Overall, for all ages and causes of calls, increased risks ranged from 9 to 16 % when comparing (study-specific) heat days or events to a reference heat measure [7–9, 17, 21], with one study reporting an increased risk of 1.45 % per degree Celsius increase in heat above a selected threshold [28]. Studies stratifying by age report all-cause increases in risk for groups as young as 15–64 [7, 16], however age-stratified effects for specific health conditions (excluding heat related illness) are generally reported for older age groups (≥65 years old) [8, 9, 16]. To date, no studies have examined EMS demand for a comprehensive array of health conditions, in a tiered system where 911 dispatch centers triage calls for basic life support (BLS) and advanced life support (ALS) response teams, or in a U.S. metropolitan area with a temperate climate where projections in climate change and vulnerability risk factors may enhance the effects of extreme heat on the population.

Both individual and regional-level factors of heat vulnerability have been identified. On an individual level, age, physical fitness, and general health have been shown to affect thermoregulation of body temperature [27, 29–31], with the very young, very old, and overweight individuals at higher risk of adverse outcomes. Regionally, the Pacific Northwest is considered to be one of the more vulnerable areas of the U.S. [32] and a stronger association between temperature and mortality has been reported in northern U.S. cities than southern U.S. cities [3]. Factors contributing to differences in regional heat vulnerability include social/environmental vulnerability (e.g. poverty), social isolation, air conditioning prevalence, and the proportion of the population that is elderly or diabetic [32]. For temperate regions, such as the Pacific Northwest, disproportional warming projections associated with climate change may exacerbate regional vulnerability by increasing the frequency and severity of extreme heat more than tropical and subtropical zones [3, 25] in an area with minimal existing heat-mitigating infrastructure (e.g. air conditioning and urban design).

The effects of extreme heat on mortality and hospitalizations have been regionally characterized for King County, WA [2, 11, 33]. With a population of nearly 2.1 million, King County is the 13th most populous county in the United States [34], accounting for approximately 30 % of the state’s population [35]. Isaksen et al. [2] demonstrated a 10 % greater, all-ages, all causes risk of death on a 99^th^ percentile heat day compared to a non-heat day, with risk of mortality “increasing 2.12 % for each degree unit increase in humidex above 36.0 °C”. Isaksen et al. [11] reported risks of hospitalization “increasing 1.59 % for each degree increase in humidex above 37.4 °C”, but no statistically significant increases in relative risk. The purpose of our research is to build on this regional assessment by analyzing EMS demand, using calls as a health metric, associated with extreme heat. We define the primary outcome as the number of basic life support (BLS) and advanced life support (ALS) incidents dispatched by the emergency dispatch centers in King County, WA for the six year period 2007–2012. Extreme heat is defined as days exceeding a humidex value, a measure of apparent temperature, which we refer to as a “heat day” throughout this paper. The increased demand for EMS calls reported in this study captures an additional heat-health burden on the population that is not likely to be reflected in the hospitalization or mortality data.

EMS of King County is a two-tiered system offering basic life support (BLS) and advanced life support (ALS). BLS is provided by firefighters who are trained as emergency medical technicians and authorized to provide non-invasive care; the average cost of these calls is $105 [36]. ALS is provided by paramedics who are authorized to administer more advanced patient care; the average cost of these calls $963 (PHSKC 2012). BLS responders are always dispatched when a medical call is placed to local 911 call centers, but ALS responders are sent only when deemed necessary. During the period 2007–2012, approximately 30 agencies responded to 165,000 BLS calls per year, while six agencies responded to 45,000 ALS calls per year [36].

## Methods

### EMS call and population data

Public Health Seattle–King County’s Emergency Medical Services Division provided the EMS call data with prior IRB approval from the University of Washington Human Subjects Division. We used a constrained calendar year, consisting of the 153 days occurring from May 1 through September 30 of each year, for a total of 918 days in the study period. Outcomes of interest included the patient’s primary health condition requiring medical assistance, known as the patient type code, and the level of transportation required by the patient.

The patient type code describes the primary health concern identified by EMS responders during a call. EMS responders use a unique coding system to identify the most likely condition of concern based on symptoms identified in the field. This system is independent of, though similar to, the International Classification of Disease codes (ICD-9 and ICD-10) that are used in hospital and mortality settings. The patient type code categories of interest include all causes, trauma, non-trauma, subcategories of non-trauma, and specific non-trauma health conditions (Table 1). All subcategories and specific non-trauma patient-type conditions were selected a priori based on the literature [2, 7, 11, 33]. Due to the nature of the EMS coding system, other variables describing the health condition of the patient were not appropriate for this analysis as they describe treatments (e.g. medication administered) or biological data (e.g. heart rate) that could be interpreted in a number of different ways. Secondary health concerns are not available in the data.

**Table 1:** Descriptive statistics for EMS data, including number of observations (n) and percent of total (%)

Variable	BLS	ALS
	*n* (%)	*n* (%)
Total calls	361,434	94,565
Average calls per day	394	103
Gender		
Male	174,667 (48)	48,779 (52)
Female	186,767 (52)	45,786 (48)
Age group		
0–4	10,436 (3)	2,141 (2)
5–14	11,414 (3)	1,654 (2)
15–44	116,587 (32)	23,194 (25)
45–64	9,887 (27)	30,426 (32)
65–84	80,221 (22)	25,407 (27)
85+	43,899 (12)	11,743 (12)
Patient type		
Trauma	47,005 (13)	6,127 (6.5)
Non-trauma	238,045 (66)	82,232 (87)
Abdominal/genito-urinary	26,452	5,172
Alcohol/drugs	17,353	4,253
Anaphylaxis/allergic reaction	3,515	1,145
Cardiovascular	30,259	24,130
Metabolic/endocrine	9,439	4,286
*Diabetes*	7,075	3,841
Neurological	47,986	13,551
*Suspected CVA*	6,432	1,354
*Suspected TIA*	694	68
*Seizures*	10,956	3,827
*Febrile seizures*	1,037	289
OBGYN	2,854	992
Other medical	58,164	14,324
*Heat illness & dehydration*	3,400	514
Psychological	18,149	3,267
Respiratory	23,874	11,112
*Asthma*	1,130	583
* Emphysema/COPD*	812	580
Not specified	76,384 (21)	6,206 (6.5)
Level of transportation	354526	93608
No transportation	87,174 (25)	12,016 (13)
Any transportation	267,352 (75)	81,592 (87)

*CVA* cerebrovascular accident, *TIA* transient–ischemic attack, *OBGYN* obstetrics/gynecology, *COPD* chronic obstructive pulmonary disease

The level of transportation required by a patient was identified as either “no transportation” or “any transportation”. We defined the latter as transportation by BLS, ALS, private ambulance, taxi, private automobile or any other mode of transportation to a hospital for further care. By including patient transportation in this study, we could observe additional measures of health severity, demand on EMS resources, and potential demand on emergency department (ED) resources.

A priori, we anticipated that several individual level characteristics might modify the effect of heat on EMS calls, including age, gender, race/ethnicity, and socioeconomic status; (the last two characteristics were not available for this dataset). Age groups of 0–4, 5–14, 15–44, 45–64, 65–84, and 85+ were created, and background population data were obtained from Washington’s Office of Financial Management [37, 38] for each age group. Calls without recorded age or gender data were excluded from the analysis.

### Meteorological-model data

The meteorological data used in this study were produced by the University of Washington’s Climate Impacts Group on the basis of the Parameter-elevation Regressions on Independent Slopes Model (PRISM) [39]. This model generated data on a grid with ~1/16th resolution (4.0 km × 7.5 km) using climate data from the National Oceanic and Atmospheric Administration’s Global Historical Climatology Network–Daily (GHCN-Daily) database [40] and knowledge of spatially relevant geographic patterns for the Pacific Northwest [41]. Each grid center contained daily values for historic temperature (minimum/maximum), humidity, and precipitation (see Isaksen et al. [2, 11] for further explanation of the meteorological models).

### Exposure assessment

Heat exposure was quantified as the average county-wide maximum daily humidex (expressed as °C). Humidex has been used as a measure of apparent temperature [2, 11, 33] and supported as an effective predictor of heat stress [42]. To calculate this heat metric, humidex was first calculated for each grid center point using the equation below (Eq. 1), the daily maximum temperature, and average relative humidity, before being averaged across King County.

1 $$ f\left(T,H\right)=T+\left( 5/ 9\right)\times \left(v- 10\right),v=\left( 6.112\times 1{0}^{\left[ 7.5T/ 237.7+T\right]}\right)\times H/ 100, $$

where T is the air temperature (°C), H is the humidity (%), and v is the vapor pressure [43]. When compared to the dry bulb temperature in King County, a region with moderate to high relative humidity, the humidex value tends to extend the extremes to reflect a higher apparent temperature on hot days and a lower apparent temperature on cooler days than the than dry temperature (Table 2).

**Table 2:** Meteorological descriptive data, 2007-2012

Meteorological data, May-September	Average county-wide maximum humidex (°C (°F))	Average county-wide maximum dry bulb temperature (°C (°F))
Minimum	5.9 (42.6)	7.1 (44.8)
Median	21.6 (70.9)	19.1 (66.4)
Maximum	44.7(112.5)	34.6 (94.3)
Meteorological data, full year	Average county-wide maximum humidex (°C (°F))	Heat days ≥ threshold (*n* (% of total days in study timeframe))
50th percentile	11.6 (52.8)	860 (93.7 %)
90th percentile	26.3 (79.3)	221 (24.1 %)
95th percentile	29.7 (85.5)	110 (12.0 %)
99th percentile	36.7 (98.1)	23 (2.51 %)
ALS optimum alert threshold	39.7 (103.5)	8 (0.87 %)
BLS optimum alert threshold	40.7 (105.3)	5 (0.54 %)

### Relative-risk analysis

Extreme heat can be defined either as a threshold temperature chosen a priori or as a percentile of previously recorded temperatures for a specific study region. Since the latter definition is generally preferred to allow for location-specific variation of effects [44], we explored the 90th, 95th, and 99th percentile of full-year humidex values and chose the model that resulted in the maximum likelihood of fit with our data for this study based on the Akaike Information Criterion (AIC) [5, 6, 33]. Days with average maximum humidex values at or above the threshold were defined as heat days; days with values below the threshold were defined as non-heat days. The relative risk of an EMS call on a heat day compared with a non-heat day was analyzed using Poisson regression and controlled for annual variation in King County’s population [3, 8]. The relative risk model equation (Eq. 2) is as follows:

2 $$ log\left({\mu}_j\ /\  population\right)={\beta}_0+{\beta}_1{I}_j\left\{ humidex> threshold\right\} $$

Where *j* indexes the day, *μ*_*j*_ is the expected call count on day *j*, and *I*_*j*_*{humidex>threshold}* is the indicator of a heat day, defined by its countywide average humidex exceeding a threshold.

### Time series analysis

We used a time series analysis to define the relationship between the intensity of heat and EMS call volume per unit increase in humidex. The analysis uses a non-parametric spline to model changes in call volume not associated with heat and a piecewise linear fit to estimate the effect of heat on calls (see Isaksen et al. [2, 11] for further explanation of this approach). The piecewise linear fit is set with two knots: one at the 50th percentile and one at the optimum alert threshold. That threshold was located by increasing the model threshold by increments of 0.1° between 25.0 and 44.7 °C humidex (the maximum humidex within the study time frame). Selection of the optimum alert threshold was based on the AIC selected, maximum likelihood of the best fit of the model. Heat intensity effects on EMS calls were estimated as the percent increase in daily EMS calls associated with a one-unit humidex increase above the optimum threshold. The time series model equation (Eq. 3) is as follows:

3 $$ {Y}_j\sim Poisson\left({P}_j{\mu}_j\right), with\  log\left({\mu}_j\right)={\beta}_0+{\beta}_1{\left({h}_j - {h}_{q 50}\right)}_{+}+{\beta}_2{\left({h}_j - {\widehat{h}}_0\right)}_{+}+s\left({t}_j\right)+{\varSigma^9}_{l= 6}{\beta}_l{I}_{\left\{ monthj=l\right\}} $$

Where *Y*_*j*_ is the observed EMS call count on day *j*, *P*_*j*_ is the population on day *j*, h_j_ is the county-wide average daily maximum humidex value on day j, h_q50_ is the 50^th^ percentile of Humidex from January 2007 through December 2012, h_0_ is the optimal alert threshold, *s (t*_*j*_*)* is the natural cubic spline modeling the overall trend of calls over 6 years, (*β*_*l*_’s) is a fixed effects adjustment for seasonal monthly effects, s (tj) is the natural cubic spline modeling the overall trend of EMS calls over 6 years, and I_month_ is the indicator variable for months May through September.

### Risk-modification factors

Two characteristics of extreme heat were hypothesized to modify the risk of an EMS call: the duration of the extreme heat [13] and the extent of the decrease in (or cooling of) the humidex overnight [45]. Duration was defined as a heat day’s position in a consecutive series of heat days; increased duration was expected to augment the effects on EMS demand. The cool-down effect was defined as the difference between average county-wide high and low humidex values for any given day above the threshold. The impact of extreme heat on health was anticipated to increase with decreasing differences between high and low humidex values as a result of this effect. Impacts of these characteristics were assessed in both the relative-risk and time series analyses.

All analyses used Oracle’s MySQL Workbench 5.2.47 CE [46] for data storage, RStudio [47] version 0.97.449 for data analysis, and Microsoft Excel [48] for table output.

## Results

Exclusion of calls with unrecorded age and gender data reduced total call counts from 441,119 to 361,434 in the BLS data and 121,974 to 94,565 in the ALS data, resulting in an average number of calls per day of 394 for BLS and 103 for ALS (Table 1). A sensitivity analysis for excluding calls with unrecorded age and gender data demonstrated that the exclusion did not significantly impact the EMS demand for all ages and all causes (two-sided *p*-value of 0.99). This reduction in total call counts did, however, reduce some already small sample sizes in some age and specific non-trauma categories to fewer than 20 calls on all heat days combined.

### Relative-risk analysis

The analysis of BLS data, representing all calls within King County, defined extreme heat as the 95th percentile (29.7 °C) of humidex, while the analysis of ALS data, representing a subset of more severe calls, defined extreme heat as the 99th percentile (36.7 °C). Of the 918 days studied, 110 fell at or above the 95th percentile and 23 fell at or above the 99th percentile (Table 2).

**Fig. 1:**
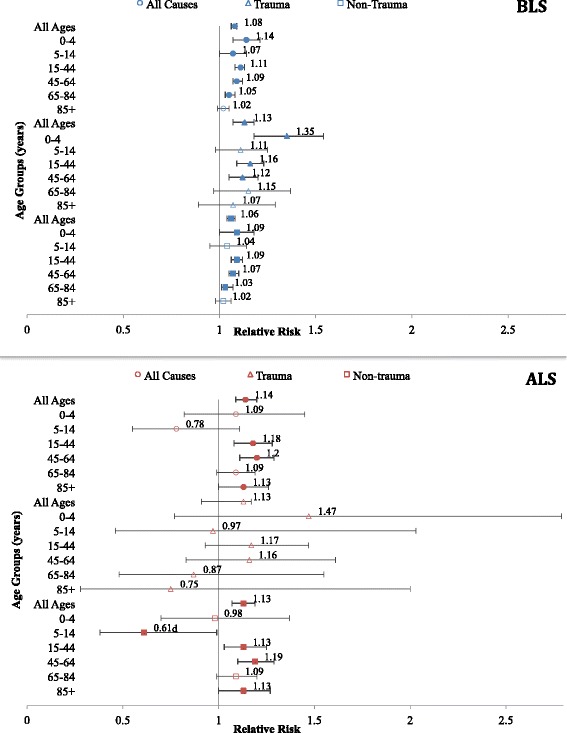
Relative risks (95 % CIs) of BLS^a^ and ALS^b^ calls for patient-type categories, by age group^c^. ^a^95th percentile (29.7 °C humidex). ^b^99th percentile (36.7 °C humidex). ^c^Solid points are significantly greater than 1 (*p <*0.05). ^d^While statistically significant, the estimate is based on a small number of cases: 1,136 cases on non-heat days, 17 cases on a heat day

Using these cutoffs, the risk of an EMS call on a heat day compared with a non-heat day increased for all causes, all ages, in both the BLS and ALS analyses (Fig. 1). The magnitude of this increase was greater for ALS (14 %, 117 vs. 103 average calls on heat days compared with non-heat days) than BLS (8 %, 420 vs. 390 average calls on heat days compared with non-heat days). Statistically significant increases in risks for all ages were also identified in both analyses for non-trauma calls, but only the BLS analysis identified increased risk of calls for trauma. For the subcategories of non-trauma-related calls, the all-ages analysis identified statistically significant increases in risk in the BLS and ALS datasets for abdominal/genito-urinary (4 % BLS, 95 % CI: 0–8 %; 23 % ALS, 95 % CI: 5–45 %), neurological (3, 95 % CI: 0–6 %; 12, 95 % CI: 12–25 %), “other medical” (17, 95 % CI: 13–20 %; 39, 95 % CI: 25–53 %), and heat illness and dehydration (243, 95 % CI: 207–284 %; 607, 95 % CI: 438–830 %) calls on a heat day compared with a non-heat day (Tables 3 and 4). The BLS analysis also revealed statistically significant increases in risk for alcohol/drug (8, 95 % CI: 3–14 %), anaphylaxis/allergy reaction (14, 95 % CI: 2–27 %), metabolic/endocrine (11, 95 % CI: 4–18 %), and diabetes (8, 95 % CI: 1–16 %) related calls. Gender was not found to affect the relationship.

**Table 3:** Relative risks (95 % CIs) for BLS^a^ data by age group and health condition*

Patient type	All ages	Age groups (years)					
		0–4	5–14	15–44	45–64	65–84	85+
All causes	**1.08(1.06,1.09)**	**1.14(1.07,1.21)**	**1.07(1,1.14)**	**1.11(1.08,1.13)**	**1.09(1.07,1.12)**	**1.05(1.03,1.08)**	1.02(0.99,1.05)
Trauma	**1.13(1.07,1.18)**	**1.35(1.18,1.54)**	1.11(0.98,1.25)	**1.16(1.09,1.23)**	**1.12(1.05,1.2)**	1.15(0.97,1.37)	1.07(0.89,1.29)
Non-Trauma	**1.06(1.04,1.08)**	**1.09(1,1.18)**	1.04(0.95,1.14)	**1.09(1.06,1.12)**	**1.07(1.05,1.1)**	**1.03(1.01,1.07)**	1.02(0.98,1.06)
Abdominal/Genito-Urinary	**1.04(1,1.08)**	1.15(0.81,1.64)	1.25(0.96,1.62)	1.04(0.98,1.11)	**1.07(1,1.14)**	0.98(0.9,1.07)	1.02(0.91,1.14)
Alcohol/Drugs	**1.08(1.03,1.14)**	1.11(0.82,1.51)	1.07(0.74,1.54)	1.06(0.99,1.13)	**1.13(1.04,1.23)**	1.01(0.84,1.2)	1.19(0.84,1.69)
Anaphylaxis/Allergy	**1.14(1.02,1.27)**	1.29(0.96,1.73)	0.93(0.67,1.28)	1.07(0.9,1.26)	**1.23(1.01,1.51)**	1.24(09.4,1.63)	1.12(0.71,1.71)
Cardiovascular	0.97(0.93,1.01)	1.61(0.92,2.83)	0.45(0.19,1.1)	0.99(0.88,1.1)	1.03(0.97,1.1)	0.93(0.87,0.99)	0.93(0.85,1.02)
Metabolic/Endocrine	**1.11(1.04,1.18)**	1.23(0.47,3.19)	1.2(0.6,2.44)	0.98(0.86,1.11)	**1.15(1.04,1.27)**	**1.18(1.05,1.32)**	**1.12(1.05,1.19)**
Diabetes	**1.08(1.01,1.16)**	0.77(0.17,3.5)	1.19(0.5,2.83)	0.92(0.79,1.07)	**1.14(1.02,1.28)**	**1.16(1.02,1.32)**	1(0.74,1.35)
Neurological	**1.03(1,1.06)**	1(0.87,1.15)	0.99(0.83,1.17)	**1.06(1,1.12)**	1.03(0.97,1.09)	1.02(0.97,1.08)	0.99(0.92,1.07)
Suspected CVA	0.97(0.89,1.05)	0.57(0.06,4.95)	--	1.2(0.85,1.71)	0.94(0.8,1.12)	0.97(0.86,1.1)	0.96(0.83,1.11)
Suspected TIA	***0.6(0.42,0.85)***	1(0.82,1.22)	1(0.82,1.22)	--	0.7(0.37,1.36)	0.64(0.4,1.02)	***0.57(0.34,0.96)*** ^***b***^
Seizures	1.01(0.95,1.08)	1.09(0.87,1.37)	0.96(0.76,1.22)	1.03(0.94,1.12)	1.03(0.92,1.15)	0.85(0.68,1.08)	0.91(0.6,1.37)
Febrile Seizures	0.96(0.8,1.15)	0.96(0.79,1.16)	1.2(0.55,2.6)	0.67(0.16,2.79)	1.64(0.56,4.76)	1.48(0.17,12.76)	0.35(0.05,2.58)
OBGYN	1.06(0.95,1.19)	0.87(0.29,2.59)	0.67(0.07,6.04)	1.07(0.95,1.2)	1.15(0.65,2.02)	0.53(0.12,2.38)	0.95(0.37,2.41)
Other Medical	**1.17(1.13,1.2)**	**1.22(1.05,1.42)**	**1.26(1.06,1.49)**	**1.24(1.18,1.31)**	**1.14(1.09,1.2)**	**1.16(1.1,1.21)**	**1.12(1.05,1.19)**
Heat Illness & Dehydration	**3.43(3.07,3.84)**	**3.89(2.08,7.29)**	**4.22(2.67,6.69)**	**4.41(3.65,5.32)**	**4.09(3.39,4.93)**	**2.91(2.52,3.37)**	**2.63(2.19,3.15)**
Psychological	1.03(0.98,1.08)	1.68(0.78,3.6)	0.99(0.72,1.34)	**1.07(1.01,1.14)**	0.99(0.91,1.08)	0.93(0.8,1.07)	0.84(0.64,1.1)
Respiratory	0.99(0.95,1.04)	0.95(0.81,1.12)	0.77(0.45,1.32)	0.86(0.68,1.09)	1.01(0.94,1.09)	1.08(0.92,1.25)	1.06(0.86,1.31)
Asthma	1.02(0.85,1.23)	1.42(0.84,2.41)	0.77(0.44,1.33)	1.1(0.83,1.46)	0.84(0.56,1.25)	1.09(0.66,1.8)	1.47(0.55,3.93)
Emphysema/COPD	0.95(0.77,1.18)	1(0.82,1.22)	--	0.82(0.1,6.38)	0.87(0.57,1.33)	1.16(0.88,1.52)	***0.41(0.18,0.96)*** ^c^

*Bolded relative risk values are significantly greater than 1 (*p <*0.05); -- indicates too few cases available to calculate

^a^95th percentile (29.7 °C humidex)

^b^While statistically significant, the estimate is based on a small number of cases [221 cases on non-heat days, 17 cases on heat days]

^c^While statistically significant, the estimate is based on a small number of cases [107 cases on non-heat days, 6 cases on heat days]

**Table 4:** Relative risks (95 % CIs) for ALS^a^ data by age group and health condition*

Patient type	All ages	Years					
		0–4	5–14	15–44	45–64	65–84	85+
All causes	**1.14(1.09,1.2)**	1.09(0.82,1.45)	0.78(0.55,1.11)	**1.18(1.08,1.28)**	**1.2(1.11,1.29)**	1.09(0.99,1.19)	**1.13(1,1.26)**
Trauma	1.13(0.91,1.17)	1.47(0.77,2.79)	0.97(0.46,2.03)	1.17(0.93,1.47)	1.16(0.83,1.61)	0.87(0.48,1.55)	0.75(0.28,2)
Non-Trauma	**1.13(1.07,1.19)**	0.98(0.7,1.37)	***0.61(0.38,0.99)*** ^b^	**1.13(1.03,1.25)**	**1.19(1.1,1.29)**	1.09(0.99,1.2)	**1.13(1,1.27)**
Abdominal/Genito-Urinary	**1.23(1.05,1.45)**	1.77(0.23,13.93)	1.51(0.21,10.81)	1.2(0.82,1.75)	1.05(0.8,1.38)	1.3(0.97,1.74)	**1.62(1.13,2.33)**
Alcohol/Drugs	1.04(0.85,1.28)	2.84(0.88,9.17)	--	0.89(0.67,1.18)	1.35(0.99,1.86)	0.83(0.34,2.05)	1.18(0.28,5.06)
Anaphylaxis/Allergy	1.06(0.73,1.54)	0.71(0.17,3.04)	0.7(0.18,2.73)	1.35(0.81,2.24)	1.16(0.58,2.31)	0.72(0.19,2.8)	--
Cardiovascular	1.02(0.93,1.12)	0.67(0.09,4.95)	--	**1.29(1.01,1.65)**	1.06(0.93,1.22)	0.97(0.84,1.12)	0.89(0.72,1.11)
Metabolic/Endocrine	1.14(0.94,1.39)	--	1.94(0.27,14.18)	1.07(0.72,1.57)	1.01(0.72,1.41)	1.29(0.93,1.79)	1.46(0.76,2.78)
Diabetes	1.12(0.91,1.38)	--	--	1.07(0.72,1.61)	1.03(0.73,1.46)	1.28(0.9,1.81)	1.19(0.55,2.56)
Neurological	**1.12(1,1.25)**	2.6(0.81,8.31)	***0.22(0.05,0.86)*** ^c^	**1.23(1,1.51)**	1.1(0.9,1.35)	1.13(0.91,1.42)	1.11(0.84,1.47)
Suspected CVA	1.27(0.93,1.74)	1(0.65,1.53)	--	1.66(0.4,6.96)	0.92(0.44,1.93)	1.22(0.73,2.03)	1.61(0.95,2.73)
Suspected TIA	2.55(0.92,7.06)	1(0.65,1.53)	1(0.66,1.53)	**20(1.9,224)** ^d^	**11.67(2.48,54.82)** ^e^	1.32(0.19,8.97)	--
Seizure	0.99(0.8,1.23)	0.81(0.36,1.83)	0.3(0.08,1.2)	1.21(0.9,1.63)	1(0.68,1.48)	0.84(0.4,1.75)	0.63(0.15,3.62)
Febrile Seizure	1.01(0.47,2.18)	1.19(0.55,2.57)	--	--	--	--	--
OBGYN	1.14(0.76,1.7)	--	--	1.17(0.79,1.75)	2.4(0.33,17.57)	--	--
Other Medical	**1.39(1.25,1.53)**	0.97(0.43,2.22)	1.16(0.45,2.99)	1.15(0.9,1.48)	**1.48(1.27,1.73)**	**1.42(1.19,1.69)**	**1.45(1.14,1.86)**
Heat Illness & Dehydration	**7.07(5.38,9.3)**	8.15(0.95,69.65)	**10.2(2.17,47.89)** ^f^	**8.6(4.7,1)** ^g^	**7.21(4.45,11.67)**	**6.68(4.36,10)**	**5.97(3.16,11)**
Psychological	1.12(0.9,1.39)	6.79(0.82,56.32)	0.95(0.12,7.2)	1(0.73,1.38)	1.33(0.95,1.85)	0.76(0.34,1.7)	1.36(0.51,3.62)
Respiratory	1.03(0.91,1.17)	0.84(0.44,1.61)	0.78(0.34,1.79)	1.08(0.79,1.47)	1.19(0.96,1.47)	0.9(0.72,1.13)	1.09(0.81,1.47)
Asthma	1.3(0.83,2.03)	2.6(0.81,8.31)	--	1.59(0.84,3.04)	1(0.31,3.3)	1.15(0.28,4.73)	1.7(0.24,12.08)
Emphysema/COPD	1.38 (0.85,2.23)	1(0.65,1.53)	--	5.81(0.72,47)	1.31(0.54,3.18)	1.38(0.74,2.6)	1.13(0.27,4.82)

*Bolded relative risk values are significantly greater than 1 (*p <*0.05); -- indicates too few cases available to calculate

^a^99th percentile (36.7 °C humidex)

^b^While statistically significant, the estimate is based on a small number of cases [1137 cases on non-heat days, 17 cases on a heat day]

^c^While statistically significant, the estimate is based on a small number of cases [379 cases on non-heat days, 2 cases on a heat day]

^d^While statistically significant, the estimate is based on a small number of cases [2 cases on non-heat days, 1 cases on a heat day]

^e^While statistically significant, the estimate is based on a small number of cases [7 cases on non-heat days, 2 cases on a heat day]

^f^While statistically significant, the estimate is based on a small number of cases [8 cases on non-heat days, 2 cases on a heat day]

^g^While statistically significant, the estimate is based on a small number of cases [71 cases on non-heat days, 15 cases on a heat day]

Analysis of subcategories of health effects by age showed statistically significant increased risks for heat illness and dehydration, as well as “other medical”, in nearly all age groups, for both datasets (Tables 3 and 4). The 15–44 and 45–64 year-old age groups experienced a statistically significant increased risk of calls for the greatest number of different types of health conditions in both the ALS and BLS analyses. Additionally, at least one age group was identified to be at a statistically significant increased risk of a call due to abdominal/genito-urinary (45–64 and 85+ year-old age groups in BLS and ALS, respectively) or neurological (15–44 year-old age group) concerns. The BLS analysis also identified statistically significant increased risks of calls for alcohol/drug (45–64 year-old age group), anaphylaxis/allergy reaction (45–64 year-old age group), metabolic/endocrine (45–63, 65–84, and 85+ year-old age groups), diabetes (45–64 and 65–84 year-old age group), and psychological (15–44 year-old age group) while the ALS analysis identified statistically significant increased risks of calls for cardiovascular (15–44 year-old age group) and suspected TIA (15–44 and 54–64 year old age groups), although the latter was based on small sample sizes. Relative small sample sizes are largely responsible for many of the wide confidence intervals in the ALS results for age group and subcategory of health effect. Effect estimates seen in the ALS data tended to be slightly higher than in the BLS data.

Decreased risks were detected in the BLS data for suspected TIA for all ages and for the 85+ age group, as well as for COPD for the 85+ age group. The ALS analysis identified decreased risks in the 5–14 year-old age group for non-trauma and neurological health effects. All decreased risks were based on small sample sizes (fewer than 20 calls on heat days), except suspected TIA for the all-ages group. Additionally, no association was found for either the 0–4 year-old age group in the ALS analysis or for the all-ages group in either analysis for cardiovascular, psychological, OBGYN, and COPD call types.

An analysis of transportation volume found an increase in no transportation (12 % BLS, 95 % CI: 9–15 %; 20 % ALS, 95 % CI: 7–35 %) and any transportation (6 % BLS, 95 % CI: 5–8 %; 14 % ALS, 95 % CI: 8–20 %) for the all-ages group on a heat day compared with a non-heat day. Similar findings are reported by age group (Table 5), with consistently greater increases in no transportation than any transportation.

**Table 5:** Relative risks (95 % CIs) of transportation of BLS^a^ and ALS^b^ calls*

	All ages	Age groups (years)					
		0–4	5–14	15–44	45–64	65–84	85+
No transportation							
BLS	**1.12 (1.09,1.15)**	**1.20 (1.09,1.32)**	1.08 (0.98,1.18)	**1.14 (1.10,1.19)**	**1.12 (1.07,1.17)**	**1.10 (1.05,1.15)**	**1.07 (1.00,1.14)**
ALS	**1.20 (1.07,1.35)**	1.46 (0.80,2.68)	0.50 (0.18,1.38)	**1.32 (1.08,1.62)**	**1.26 (1.04,1.53)**	1.06 (0.83,1.36)	1.13 (0.81,1.59)
Any transportation							
BLS	**1.06 (1.05,1.08)**	**1.11 (1.02,1.20)**	1.05 (0.97,1.14)	**1.09 (1.06,1.12)**	**1.08 (1.05,1.10)**	**1.04 (1.01,1.07)**	1.00 (0.97,1.04)
ALS	**1.14 (1.08,1.20)**	1.02 (0.75,1.40)	0.86 (0.59,1.24)	**1.14 (1.04,1.26)**	**1.20 (1.10,1.30)**	**1.09 (1.00,1.20)**	1.12 (0.99,1.27)

*Bolded relative risks are significantly greater than 1 (*p <*0.05)

^a^95th percentile (29.7 °C humidex)

^b^99th percentile (36.7 °C humidex)

### Time series analysis

The time series Humidex temperature thresholds of extreme heat were defined as 40.7 °C for BLS (5 of 918 days) and 39.7 °C for ALS (8 of 918 days) data on the basis of model best fit.

This study found an increase—6.6 % (95 % CI: 4.5–8.7 %) for BLS and 3.8 % (95 % CI: 1.09–6.5 %) for ALS—in EMS calls for each one-unit increase in humidex above the analyses’ respective thresholds for all causes, all ages (Table 6). Increased risks per unit increase in humidex were also identified in all ages for non-trauma (10 % BLS, 95 % CI: 7.6–12.5 %; 4.2 % ALS, 95 % CI: 1.3–7.1 %), other medical (23.5 % BLS, 95 % CI: 19.6–27.4 %; 18.3 % ALS, 95 % CI: 12.9–23.9 %), heat and dehydration (48.5 % BLS, 95 % CI: 39.9–57.7 %; 48.9 % ALS, 95 % CI: 35.9–63.1 %), neurological (5.9 % BLS, 95 % CI: 1–11.1 %), and psychological (11.5 % BLS, 95 % CI: 4.1–19.4 %), but not by gender. When stratifying by age group, we found significant increased risks of BLS calls with sufficiently large sample size (greater than 20 calls on all heat days) in all causes (0–4, 15–44, 45–64, 65–84, and 85+ year-old age groups), non-trauma (5–14, 15–44, 65–84, and 85+ year-old age groups), neurological (45–64 year-old age group), other medical and heat and dehydration (15–44, 45–64, 65–84, and 85+ year-old age groups). For ALS, increased risks with sufficiently large sample size for age groups include all causes (45–64 year-old age group) and other medical (15–44, 45–64, and 65–84 year-old age groups). Subcategories of patient-type conditions were also associated with increased risk in BLS and ALS data stratified by age, however all were based on fewer than 20 calls on heat days. The only call type resulting in a reduced risk was trauma (−11.5 %, 95 % CI: (−20)–(−2.6)) in the BLS 15–44 year-old age group.

**Table 6:** Increased risk (95 % CIs) per unit humidex increase above threshold for BLS^a^ and ALS^b^ analyses*

	All ages	Age groups (years)					
		0–4	5–14	15–44	45–64	65–84	85+
All causes							
BLS	**6.6 (4.5,8.7)**	**11.6 (1.7,22.4)**	4.7 (−5.5,15.9)	**5.1 (1.9,8.3)**	**8.6 (5.3,12.1)**	**7.6 (3.7,11.7)**	**8.1 (2.9,13.6)**
ALS	**3.8 (1.09,6.5)**	4.2 (−11.1,22.2)	2.1 (−16.8,25.2)	1.9 (−3,7)	**7.7 (3.5,12.0)**	2.3 (−2.7,7.5)	3.6 (−3.5,11.2)
Trauma							
BLS	−4.3 (−10.2,2.0)	3.4 (−17.2,29.1)	14.5 (−33.9,10.6)	***−11.5 (−20,−2.6)***	−2.9 (−13.1,8.5)	4.5 (−7.1,17.6)	7.0 (−8.1,24.7)
ALS	−6.3 (−16.5,5.3)	24.7 (−5.8,65.2)	−4.7 (−44.8,64.4)	−8.7 (−21.9,6.7)	−8.5 (−28,16.3)	−18.9 (−51,34.0)	−2.1 (−45.3,75)
Non-trauma							
BLS	**10.0 (7.6,12.5)**	−0.5 (−100,Inf)	**7.9 (4.5,11.5)**	**10.9 (6.2,15.8)**	−0.5 (−100,Inf)	**7.9 (4.5,11.5)**	**10.9 (6.2,15.8)**
ALS	**4.2 (1.3,7.1)**	−7.1 (−26.1,16.9)	−1.3 (−24,28.2)	1.7 (−3.9,7.6)	7.8 (3.5,12.3)	2.2 (−2.9,7.5)	4.2 (−2.9,11.8)
No transportation							
BLS	**10.9 (7.30,14.6)**	**19.2 (5. 6,34.5)**	**18.0 (3.7,34.3)**	**7.2 (1.8,12.9)**	**16.0 (9.8,22.6)**	**8.2 (0.7,16.2)**	7.6 (−2.4,18.7)
ALS	**10.0 (3.8,16.6)**	15.7 (−15.3,57.9)	10.9 (−22.1,58.1)	**12.8 (3.0,23.5)**	**12.8 (3.0,23.5)**	3.0 (−10.6,18.5)	−4.1 (−24.2,21.2)
Any transportation							
BLS	**5.6 (3.3,8.0)**	2.9 (−10.2,17.8)	−4.2 (−17.2,11.0)	**3.8 (0.1,7.6)**	**6.3 (2.4,10.3)**	**7.4 (3.1,11.9)**	**8.1 (2.2,14.2)**
ALS	**3.1 (0.2,6.1)**	−2.1 (−19.5,19.2)	−1.0 (−22.4,26.3)	−1.3 (−6.8,4.4)	**6.9 (2.4,11.6)**	2.0 (−3.3,7.6)	4.6 (−2.8,12.6)

*Bolded estimates are significantly greater than 1 (*p <*0.05)

^a^40.7 °C humidex threshold

^b^39.7 °C humidex threshold

An analysis of transportation volume identified increases in both no transportation (10.9 % BLS, 95 % CI: 7.3–14.6 %; 10.0 % ALS, 95 % CI: 3.8–16.6 %) and any transportation (5.6 % BLS, 95 % CI: 3.3–8.0 %; 3.1 % ALS, 95 % CI: 0.2–6.1 %) for the all-ages group per degree increase in humidex above the respective optimal thresholds. Similar findings are reported for the 15–44, 45–64, and 64–85 year-old age groups (Table 6), with consistently greater increases in no transportation than any transportation.

### Risk-modification factors

Analysis of the temporal characteristics of heat duration and nighttime cooling identified significant, though minimal, changes in the effect estimates for the BLS relative risk analysis only with increasing duration and increasing nighttime cooling each resulting in higher risks. The most frequent heat duration during the study timeframe was 1 day (13 occurrences), followed by 2 days (11 occurrences), with a maximum of 9 days (one occurrence in 2009). For all ages, all causes, there was an estimated change in calls of 0.01 calls per day of added duration. While this finding is statistically significant, the increase is arguably very small and possibly attributable to the observed increase in maximum daily average county-wide humidex during multi-day exposures.

The analysis of cool down assessed for a change in effect associated with a change in average daily extremes (daytime high to nighttime low humidex). During the study timeframe the range in the difference between average daily high and low humidex was 14.03 to 28.71 °C, with the difference increasing as average daily maximum humidex increased. The estimated change in all age, all cause BLS calls was 0.013 per degree increase in daily humidex difference (SE 0.003), demonstrating an increasing risk of an adverse health effect with increased cooling. However, this increase is also arguable very small. It should also be noted that the average daily minimum humidex also increased as the maximum humidex increased. As a result, the increased risk demonstrated by increased cooling may be the result of higher overall temperatures rather than an increase in the difference in high to low temperatures. Future analyses should more thoroughly investigate the diurnal trends in humidex and their association with call volumes to assess whether this relationship changes under certain conditions.

## Discussion

This study offers a comprehensive analysis of health conditions necessitating emergency medical services stratified by age groups. It assesses the impacts on BLS and ALS levels of care independently and contributes to the limited body of literature characterizing changes in EMS transportation volume. Moreover, this study contributes to the ongoing characterization of the effects of extreme heat on health for a region of the United States that is highly vulnerable to such effects [32]. It also advances the discussion about the reportedly stronger association between temperature and mortality in northern U.S. cities versus southern U.S. cities [3].

Our overall findings—an increased risk of an EMS call for all ages and all causes of 8 % for BLS and 14 % for ALS—demonstrate a significant effect of heat that is consistent with the 9–16 % range reported in existing EMS literature [7–9, 17, 21] and previous findings for mortality (10 % increase on a 99th percentile heat day compared with a non-heat day) within King County, WA [3]. While the observed BLS relative risk effect estimates were consistently lower than the ALS estimates, the narrower confidence intervals and larger sample sizes for the age groups and specific health conditions allows us to be more confident in the result of the BLS analysis. The higher model-selected humidex threshold for the ALS analysis (99th percentile versus 95th percentile) likely contributed to the larger effect estimates in the ALS analysis of relative risk compared with the BLS analysis. This explanation is supported by the reversal in the relative magnitude of the optimum thresholds and the reported effects in the time series analyses, where the BLS data demonstrated a 6.6 % increase in risk per unit humidex increase above 40.7 °C and the ALS data demonstrated a 3.8 % increase in risk per unit humidex increase above 39.7 °C.

A central finding of this study is the high frequency of increased relative risks across health conditions for the 15–44 and 45–64 year-old age groups. While consistent with the limited EMS and heat literature stratified by a similar level of age specificity [7, 16] and recent findings in emergency department visits [10, 15], these results indicate vulnerability in an age range generally considered to be relatively resilient, and for which no effect has been demonstrated in analyses of other regional health-surveillance data [2, 11]. Since the working-aged population comprises these two age groups, these increased risks may reflect hazards related to occupational or recreational activities, such as inadequate hydration or rest [49], or differences in risk factors, such as obesity, that affect one’s ability to thermoregulate during periods of exertion [29]. We were unable to consider these potential contributors to risk in our study because the EMS data does not contain variables describing body mass or indicating whether a call was related to work or recreational activities. General differences in body size, activities, and occupations associated with gender may also be informative of the risk factors leading to increased risks in these age groups, however gender was not found to affect the relationship of heat and EMS calls.

The presence of statistically significant results in the 0–4 and 5–14 year-old age groups is also an important finding in our study that corroborates existing literature demonstrating increased risk of morbidity, mortality, and emergency department visits in children associated with heat exposure [12, 15]. Children are commonly considered to be a vulnerable population, yet research demonstrating an association between child health and heat is limited [9, 12, 15, 21]. In this study we found significant increased risks of a BLS call in 0–4 and 5–14 year-old age groups from all causes, “other medical”, and heat and dehydration as well as trauma and non-trauma in the 0–4 year-old age groups, despite the relatively small sample sizes for children. Considering how other recent studies on the effects of heat on mortality and hospitalizations in King County do not report increased risks for these age groups, our findings indicate that the effects of heat on child health may be captured at an EMS level, but not higher up in the public health system [50].

When stratified by health condition, the results revealed at least one age group at significantly increased risk for eight of the non-trauma patient type categories and three of the specific health conditions. As expected, the greatest increases in risk were attributable to heat illness and dehydration, with risks increasing by as much as 341 % in BLS calls (15–44 year-old age group) and 621 % in ALS calls (45–64 year-old age group) on a heat day compared with a non-heat day. All ages heat illness and dehydration calls increased by 48.5 % for BLS and 48.9 % for ALS calls per-unit humidex increase above their respective thresholds. These rates exceeded those reported in comparable analyses: 43 % for heatstroke [20] and 29 % for heat-related illness [18]. Interestingly, heat illness and dehydration were the only commonly assessed health conditions in the heat-health literature to be associated with consistent increases in risk across age groups in this study. Increases in risk for cardiovascular and respiratory illnesses, the other two commonly included categories, were not significant except in the ALS relative-risk analysis of the 15–44 year-old age group for cardiovascular events; but we identified significant health conditions previously not included in the EMS heat-health literature.

Previous studies have examined the effect of extreme heat on patients experiencing neurological conditions, diabetes, and psychological issues. Neurological conditions were assessed in an Australian study of heat-related impacts on EMS by all causes as well as cardiovascular and respiratory conditions, but statistically significant neurological results were not reported [7]. Heat was demonstrated to increase the risk of diabetic-related mortality in the 45–64 year-old age group in the Pacific Northwest—one of the age groups (along with the 65–84 year-old age group) identified in this study as being at increased risk of diabetes [2]. Increased risks of psychological conditions have also been reported in relation to extreme heat [51], although not for EMS calls. The remaining associations revealed in this study—abdominal/genito-urinary, alcohol/drug, and anaphylaxis/allergy reaction—have received no mention in relevant literature.

When stratified by transport activity, this study’s results revealed that the directional agreement of increases in no transportation and any transportation across age groups and analyses reflects an overall increase in calls, but no clear change in the composition of patient transportation needs. However, the relatively greater increase in the no transportation category suggests a greater proportion of the heat-related calls were treatable by EMS in the field since they did not require transportation to an Emergency Department. This finding is contrary to the 24.8 % overall increase in transportation on heat days compared with non-heat days reported for EMS in Boston, Massachusetts [21]. These opposing results could be attributable to differences between the model used in this study (threshold of extreme heat) and the one used by Kue and Dyer (regionally accepted trigger for heat warnings); the inclusion of rural calls in King County versus restriction to urban calls in Boston; or regional differences in population and access to health care. In any case, EMS transportation (an activity resulting in emergency department visits) provides insight into the impact on emergency departments—a component of regional health-surveillance systems in King County that currently lacks comprehensive data.

The high optimum thresholds selected by the time series analyses introduced tension between the use of established statistical methodology and the potential integration of results into practice. The method used to select these thresholds, the Akaike Information Criterion is commonly applied in statistical analyses, but the high thresholds of 40.7 °C humidex for BLS and 39.7 °C humidex for ALS—both considerably greater than even the 99th percentile of humidex (36.7 °C)—not only resulted in an analysis based on an extremely limited sample size but also produced risk estimates that would be applicable only a few days per year. Since these high thresholds were driven by one heat wave in 2009, expansion of the study time frame would likely attenuate them to a more relevant level for current management efforts and potential policy applications. However, thresholds of this intensity are arguably not protective of vulnerable populations or appropriate for early warning systems as they represent the most extreme exposures for this region. The researchers did investigate alternative thresholds with more promising policy and management applications by forcing the model to use the 99^th^ percentile (36.7 °C), but effect estimates were generally unchanged or slightly weakened. While the high thresholds selected by the AIC are statistically appropriate and may be indicative of future trends in extreme heat—and thus should not be completely discounted—further investigation into the most appropriate approach to setting time series thresholds should be considered to maximize both the scientific and practical applications of climate change and human health research.

### Strengths and limitations

This study has several limitations. We excluded approximately 20 % of records that were missing information on age or gender. This may have affected our ability to detect true increases in EMS call volume, especially when stratifying by age or call type due to diminished study power. However, a sensitivity analysis found that the associations we report for all ages and all causes would not have changed if we had included all records in the analysis.

Accurate representation of King County’s EMS needs could have been influenced by the use of private ambulance services if the distribution of calls made directly to those services, and thus bypassing 911 dispatchers, caters disproportionately to particular age groups, such as those in retirement facilities. Future studies should attempt to collect data from private EMS services in addition to public services.

Misclassification of disease may have occurred as a result of inherent differences between the patient type codes used by EMS responders and the ICD codes that are the gold standard for physician diagnoses. EMS responders identify the most likely condition of concern on the basis of symptoms observed in the field with the limited time and tools available to them. Since some conditions present with similar symptoms, it is possible for the assessment of a given health condition to change in an emergency department setting, where additional diagnostic criteria may be applied. Nonetheless, the study’s results are still highly applicable to public health interpretation as well as to EMS planning and preparation because they describe patients’ physical states during periods of extreme heat.

The county-wide averaging of meteorological data, use of average relative humidity, and reliance solely on outdoor conditions likely introduced exposure misclassification to the analysis. The 5,480 km^2^ region of King County [35] includes a range of climatological, geographic, and demographic areas, such as densely populated, sea-level cities on the western edge (where the humidex tends to be slightly higher), to rural, forest-covered mountains in the east (where humidex tends to be slightly lower). Averaging conditions across these differences likely attenuated the daily maximum humidex for this County’s primarily western-centered population, resulting in misclassification of calls as unexposed (type 2 errors). An alternative approach, in which each EMS call is spatially assigned to the nearest meteorological-grid center point, rather than averaging the calls across the region, should be considered for future research. Exposure misclassification may have resulted from the use of average relative humidity, the only available form of relative humidity in the meteorological data, in the calculation of maximum humidex. While the heat-health relationships reported in this study should not have been affected, the thresholds may be slightly inflated due to the inverse relationship of temperature and relative humidity. The inability to capture conditions that contributed to, or mitigated, individual-level exposures—including access to air conditioning or shade—may have introduced additional exposure misclassification that the data could not address.

We did not adjust for the presence of multiple comparisons in this study primarily because the nested nature of the outcome variables facilitated a staged analysis where significant impacts on all ages and all causes were identified prior to investigating specific age groups or subcategories of health. Additional justification for not adjusting the analysis is rooted in study’s aim to include a hypothesis-driving component for regional planning and preparedness efforts as well as for future research into the effects of heat on health. While a Bonferroni correction would have reduced the potential for type 1 error, it may also have increased type 2 error—the presence of which eliminates the hypothesis-driving aspects of the study and has negative implications for practitioners aiming to mitigate the impacts of heat on health. Rather than shift the error type from 1 to 2, we opted to present all study results, highlighting those associated with a small sample size (≤20 calls) or inconsistencies.

## Conclusions

This study demonstrated a positive association between extreme heat and EMS call volume in King County, WA that is consistent with the existing EMS literature and regional effects demonstrated for other health outcomes. The high frequency of age-specific effects in working aged adults (15–44 and 45–64 year-old age groups), as well as health-specific effects not previously studied in related literature (abdominal/genito-urinary, alcohol/drug, and anaphylaxis/allergy reaction), indicate that ongoing characterization of heat-related health effects and regional climate change–adaptation vulnerability should consider expanding their scope to be more comprehensive. The presence of effects in children (0–4 and 5–14 year-old age groups) not reported in other regional studies of heat on mortality and morbidity adds important insight into our understanding of how this vulnerable population is represented in the public health literature. The overall finding that increases in calls necessitating transportation as well as calls treatable on scene may provide valuable guidance for public planning and management after further investigation is completed. Future research should concentrate on the limitations of the county- and day-level exposure assessment as well as activity-based risk factors.

## Abbreviations

ALS: advanced life support
BLS: basic life support
COPD: chronic obstructive pulmonary disease
CVA: cerebrovascular accident
EMS: emergency medical services
OBGYN: obstetrics/gynecology
TIA: transient-ischemic attack
